# Dramatic Radiographic Response of Pelvis-Filling Locally Advanced Cervical Cancer Treated With Radiation and Chemotherapy

**DOI:** 10.7759/cureus.61544

**Published:** 2024-06-02

**Authors:** Mannat Bedi, Aria Kieft, Michael Joiner, Steven Miller

**Affiliations:** 1 Department of Oncology, Wayne State University School of Medicine, Detroit, USA

**Keywords:** concurrent chemoradiation therapy, hdr (high dose rate) brachytherapy, gynecology-oncology, intensity modulated radiation therapy (imrt), locally advanced cervical cancer

## Abstract

Locally advanced cervical cancers are often treated with palliative intent due to concerns that the tumor is too far advanced or too large to be treated curatively. Also, patients greater than 65 years of age with cervical cancer are sometimes regarded as being too old or too frail to be cured with combined radiation and chemotherapy. These patients are often treated with radiation alone or with palliative therapy. Understanding the treatment modalities for cervical cancer is essential, as they can be complex and unique to each patient's specific diagnosis. This case report aims to describe the dramatic response to treatment with combined radiation and chemotherapy for a patient greater than 65 years of age with pelvis-filling cervical cancer with right-sided hydronephrosis. After a five-week course of concurrent chemoradiation, the cervical mass radiographically completely disappeared, with no evidence of disease noted on pelvic MRI.

## Introduction

The incidence and mortality of cervical cancer in the U.S. has been declining, but it remains the second leading cause of cancer deaths for women between 20 and 39 years of age [1]. In 2020, 11,542 new cases of cervical cancer were reported in the United States [2-3]. The incidence of cervical cancer in older women has remained relatively unchanged, with a quarter of the cases occurring in women after the age of 65 years. These patients often present with later stages of disease [4]. Currently, a combination of surgery, chemotherapy, external beam radiation therapy, brachytherapy, as well as immunotherapy have been used to treat cervical cancer patients [5]. For the treatment of locally advanced cervical cancer, concurrent chemoradiation therapy, which consists of cisplatin chemotherapy and external beam radiotherapy together, followed by an intracavitary or interstitial brachytherapy implant and immunotherapy, is now the standard of care [5]. The use of advanced imaging, including MRI and positron emission tomography (PET) scans, for staging purposes and to evaluate response to treatment has also become increasingly important in treating cervical cancer [6]. This was especially demonstrated in this case report, in which a patient greater than 65 years old with pelvic-filling cervical cancer had a complete radiographic response to concurrent chemoradiation therapy with no evidence of disease noted on imaging before the initiation of brachytherapy.

## Case presentation

Our patient is a 69-year-old female with a past medical history of GI bleeding, cholecystitis, hypertension, arthritis, anemia, aortic stenosis, and tricuspid valve regurgitation who presented to the clinic with a six-month history of vaginal bleeding. She is also a non-smoker and non-drinker with a past gynecology history significant for seven vaginal deliveries.

**Figure 1:**
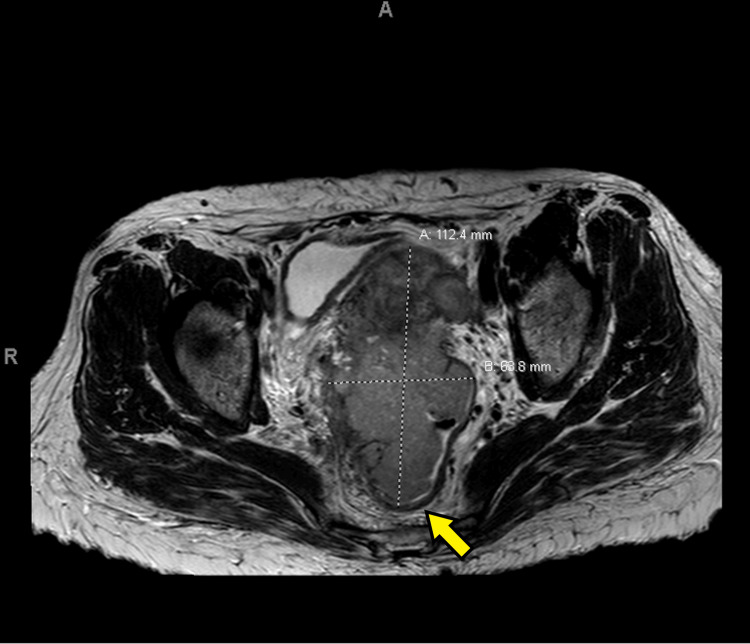
Axial MRI of the cervical lesion at diagnosis The tumor measures approximately 11.2 cm in AP x 6.8 cm in axial dimension. The arrow points at the area of possible rectal involvement.

**Figure 2:**
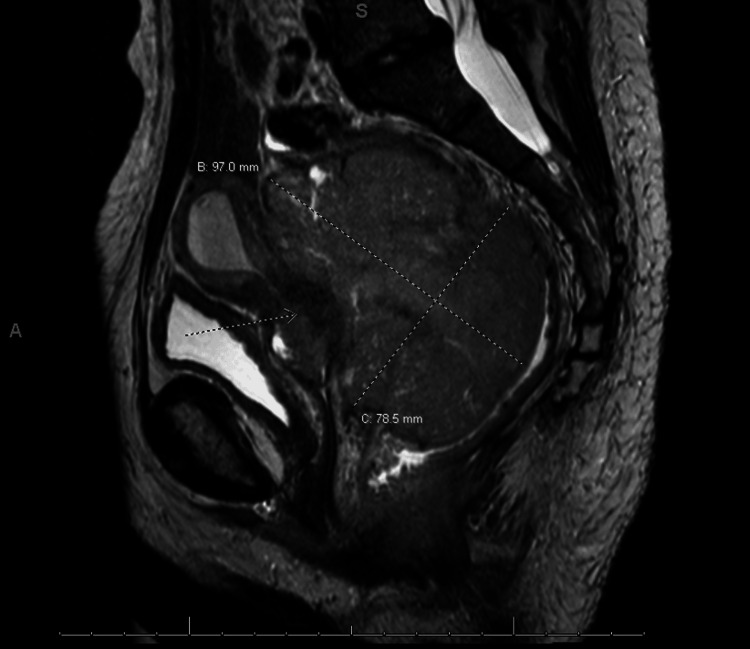
Sagittal MRI at diagnosis reveals a large pelvic mass measuring 9.7 cm by 7.8 cm, compressing the uterus, rectum, and bladder The arrow is pointing to what remains of the normal cervix.

She underwent an evaluation, including a pelvic exam, which revealed a large fixed mass involving the cervix with bilateral pelvic sidewall involvement and minimal vaginal involvement. A biopsy of the cervical mass was performed, and pathology was consistent with a poorly differentiated non-keratinizing squamous cell carcinoma. Staging studies, including a CT scan of the abdomen and pelvis, as well as an MRI of the pelvis, revealed a lesion involving the cervix measuring 11.2 x 6.8 x 9.5 cm in size. No enlarged pelvic or per-aortic lymph nodes were noted, but minimal right-sided hydronephrosis was seen, as well as possible involvement of the rectal wall noted on MRI. (Figures 1-2) A rectal biopsy was performed, and pathology was negative for a malignancy, so she was staged as a IIIB cervical cancer. Her Karnofsky performance status was 80.

**Figure 3:**
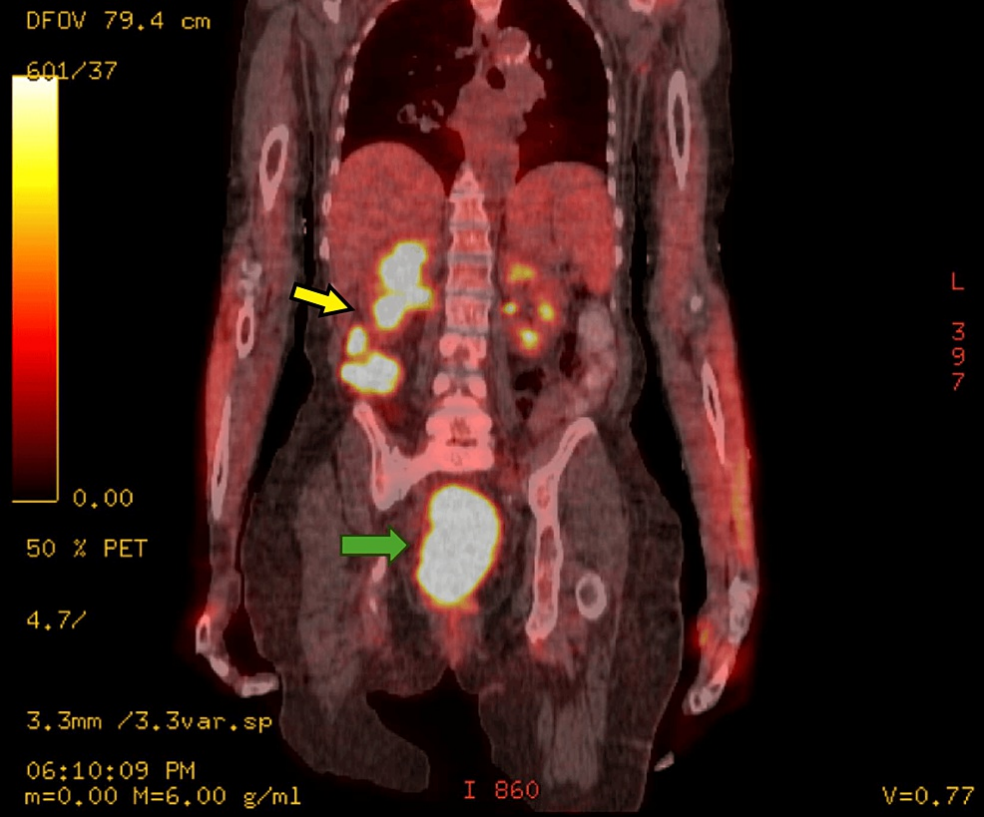
PET scan with a green arrow pointing to the large cervical mass No involved lymph nodes were noted on PET. The yellow arrow points to the physiological uptake seen in the right kidney and bowel, which is not consistent with the disease. PET: positron emission tomography

Additional staging studies were obtained, including a PET scan, which revealed a large F-18 fluorodeoxyglucose (FDG)-avid pelvic mass, demonstrating a maximal standardized uptake value (SUV) of 14.1 (Figure 3). No pelvic or para-aortic lymph nodes were FDG-avid.

**Figure 4:**
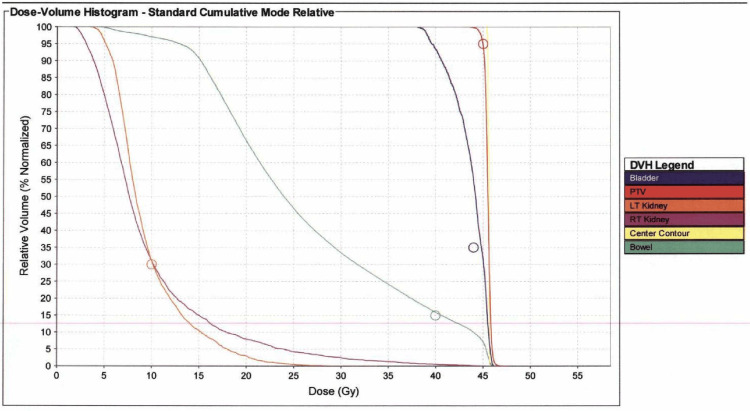
Dose-volume histogram (DVH): the planning target volume (PTV) is in red, the bladder is in black, and the right and left kidneys are purple and orange. The bowel is contoured in blue.

She underwent a course of primary radiation and chemotherapy to the pelvis and para-aortic lymph nodes; the para-aortic lymph nodes were treated secondary to a greater than 15% risk of their involvement with microscopic disease. A planning CT scan without contrast using 3 mm slices was obtained for radiation planning. A pelvic cradle was fashioned prior to the planning CT scan to assist with positioning and a CT scan with the bladder full and empty was obtained prior to initiation of treatment to evaluate the movement of the uterus. The gross tumor volume (GTV) consisted of the cervix and the cervical disease as seen on MRI. The clinical target volume (CTV) included the cervix and the cervical lesion as seen on MRI with a 1 cm expansion plus the uterus, parametria, proximal vagina, and pelvic and para-aortic lymph nodes. The planning target volume (PTV) was an approximate 1 cm expansion around the CTV. She was treated using intensity-modulated radiation therapy (IMRT) to the pelvis and para-aortic lymph nodes. She received a total dose of 4500 cGy in 25 fractions to the pelvis and para-aortic lymph nodes. The PTV received a total dose of 4500 cGy to approximately 95% of the volume and less than 10% of the right and the left kidney received a dose of greater than 1800 cGy (Figure 4).

**Figure 5:**
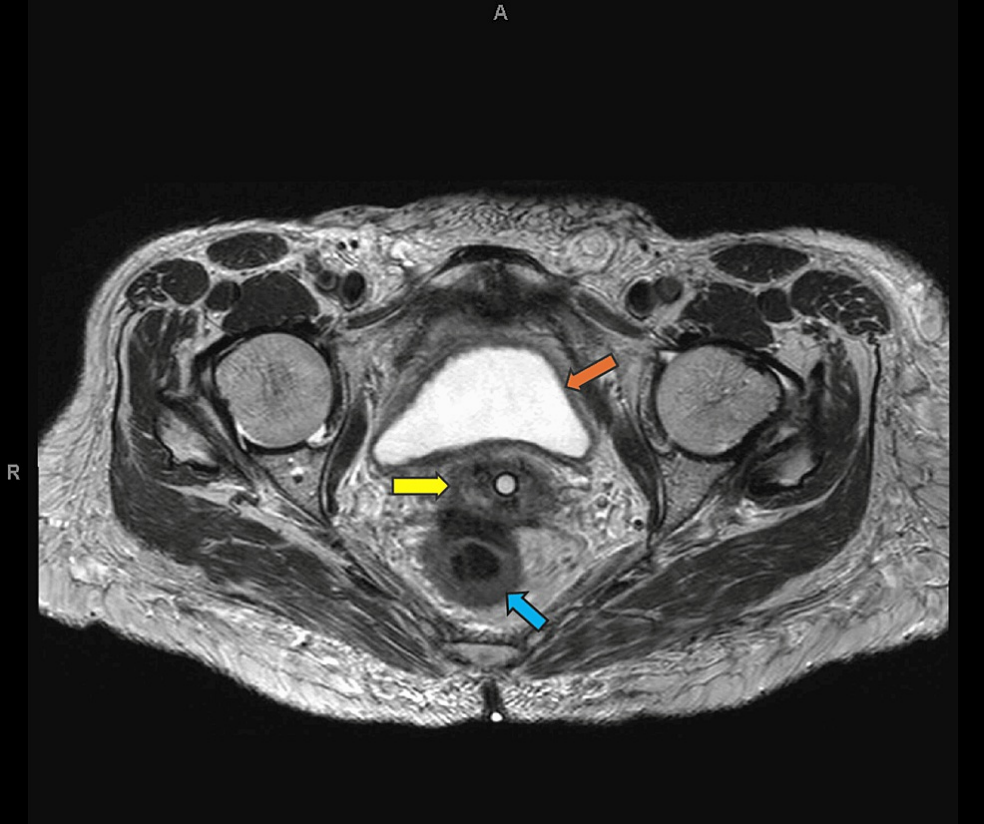
Axial MRI showing complete response to therapy with no radiographic evidence of the cervical mass Orange arrow: bladder, Yellow arrow: Cervix with no disease seen, Blue arrow: Rectum

**Figure 6:**
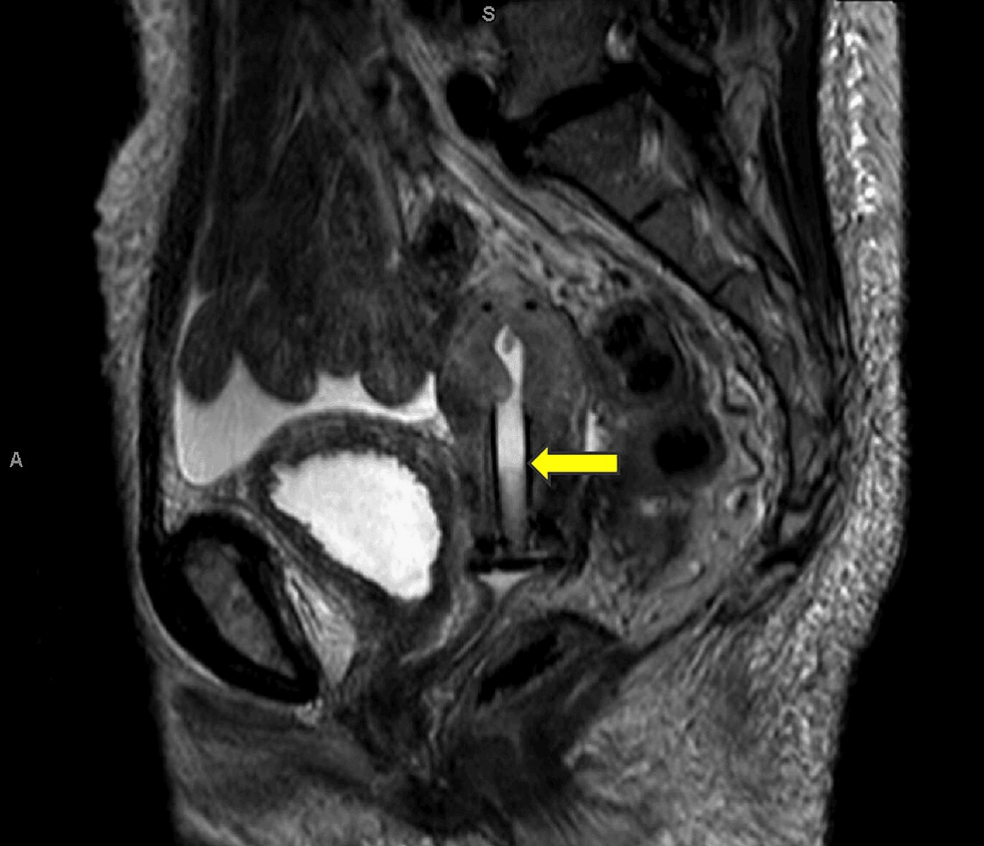
Sagittal MRI showing complete radiographic response after radiation and chemotherapy The cervical cancer is not visible but a Smit sleeve that was placed through the cervical os and into the uterus for brachytherapy is present (yellow arrow).

The patient responded well to the radiation and chemotherapy regimen and had limited side effects. The only side effects included Radiation Therapy Oncology Group grade one gastrointestinal toxicity consisting of decreased appetite with less than 5% weight loss. A follow-up CT scan of the chest, abdomen, and pelvis revealed no evidence of metastatic disease. An MRI of the pelvis obtained approximately five weeks after the initiation of radiation and chemotherapy demonstrated a dramatic decrease in the size of the pelvic mass, with a complete radiographic response of the tumor noted. No evidence of disease was reported in the cervix, and only the Smit sleeve, which had been placed for the brachytherapy procedure, was seen in the cervix (Figures 5-6).

**Figure 7:**
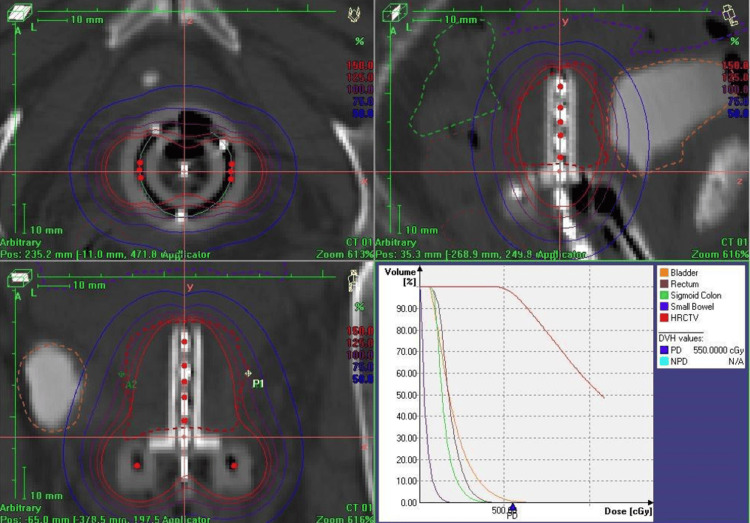
HDR brachytherapy treatment showing the tandem and ring in the axial, sagittal, and coronal planes; a dose volume histogram (DVH) displaying the dose to the organs at risk

She then underwent 5 high-dose-rate (HDR) brachytherapy treatments to a total dose of 550 cGy x 5 to greater than 90% of the high-risk CTV (HRCTV) after the completion of 5 weeks of external beam radiation therapy (Figure 7). The HRCTV was defined as the visible cervix on MRI imaging plus any gross disease after external beam irradiation. The total combined biological equivalent dose (BED) to the HRCTV using an alpha over beta of 10 for the tumor was 95 Gy, the BED to 2 cc of the bladder using an alpha over beta of 3 for organs at risk (OARS) was 78.9 Gy, the BED dose to 2 cc of the rectum was 59.1 Gy, and the BED to 2 cc of the sigmoid was 62.6 Gy. She completed her course of treatment in just under 10 weeks. Her treatment was slightly delayed, secondary to transportation issues (Figure 7).

The patient overall recovered well from radiation and chemotherapy but, unfortunately, nine months after treatment, she presented to the emergency department secondary to weakness, fatigue, nausea, vomiting, and constipation, and was found to be in acute renal failure. A CT scan of the abdomen and pelvis at that time revealed distension of the small intestine, which was suspicious for a partial mechanical obstruction and some abdominal ascites. No pelvic or cervical masses, recurrent disease, or evidence of hydronephrosis was noted on imaging.

Stool softeners and Lactulose were initiated upon admission for suspected ileus. However, her condition worsened as well as her gastric distension. A nasogastric tube was placed and her white blood cell count increased significantly. Secondary to her cardiac comorbidities, she was transferred to the intensive care unit (ICU). Due to her metabolic acidosis, she was intubated in the ICU and was found to have gram-negative bacteremia and was treated per sepsis guidelines. She continued to develop a rigid abdomen with a high suspicion of perforation. She remained unresponsive on vasopressors. She was converted to comfort care and expired shortly afterward.

## Discussion

Globally, 59 years of age is the average age of death for women with cervical cancer. Older women, 60 or greater, are more likely to be diagnosed with advanced-stage disease. Women with stage III-IV disease have higher recurrence rates and worse survival, with a five-year survival of 39.7%-41.5% for stage III disease and 22% for stage IVA disease [7].

In the initial evaluation of many of these patients with cervical cancer, staging studies, including PET and MRI scans, assist in determining the extent of the cervical disease as well as any nodal or distant involvement of the disease [8]. Imaging is also helpful in evaluating a patient's response to treatment. MRI imaging is essential for brachytherapy treatment planning after external beam radiation therapy, in which the radiation treatment is tailored to treat the cervix as well as any residual disease that can be easily seen on MRI.

Cervical cancer is a complex disease entity with multiple treatment options available to the patient, including surgery, radiation, chemotherapy, and immunotherapy [5]. Yang et al. performed a retrospective survival analysis of 44,602 patients using the Surveillance, Epidemiology, and End Results (SEER) database. The results of this analysis demonstrated that if primary surgery for cervical cancer patients is not performed, treatment with radiation therapy is of the most benefit [9]. Radiotherapy for later-stage disease, stages III-IV, can improve overall survival and cause-specific survival for cervical cancer patients [9]. Yang et al. also found on multivariate analysis that radiotherapy had a beneficial effect on patients with cervical cancer who were peri or post-menopausal but found an adverse impact in pre-menopausal women, showing that age around 45 years may play a role in the effectiveness of therapy. Multivariate analysis also revealed that radiotherapy for tumors greater than 3 cm was beneficial while radiotherapy for less than 3 cm tumors was harmful [9]. Finally, In addition, combined external beam and brachytherapy were more effective in treating cervical cancer patients than just external beam radiation alone.

According to the American Society for Radiation Oncology (ASTRO) guidelines from 2020, chemoradiation is the recommended treatment option for stages IB3-IVA cervical cancer [10]. Further, Chino et al. noted that for definitive radiation therapy, radiation doses can be administered from 4500 to 5040 cGy, in 180 cGy fractions to the pelvis, along with weekly cisplatin and intra-cavitary or interstitial brachytherapy with the completion of radiation therapy in eight weeks or less [10]. Further, guideline recommendations note that brachytherapy for locally advanced cervical cancer should not be replaced with IMRT or stereotactic body radiation therapy (SBRT), as many studies have pointed out the association between brachytherapy and increased survival compared to IMRT and SRBT [10]. Gill et al. noted that omitting brachytherapy negatively affects survival outcomes more strongly than excluding chemotherapy [11].

Providing brachytherapy is the standard of care for treating locally advanced cervical cancer [10]. Other case reports similar to the one presented have found mixed results regarding response to treatment. In a report by Bhagat et al., a 69-year-old female with International Federation of Gynecology and Obstetrics (FIGO) stage IIA2 cervical adenosarcoma with a lesion size of 9x 6x 5 cm occupying the cervix and lower uterine segment was non-responsive to chemoradiation [12]. This patient received 4800 cGy in 24 fractions of external beam radiation therapy and carboplatin weekly with a boost to the cervical mass of 1400 cGy in seven fractions [12]. The patient did not undergo brachytherapy. After the completion of treatment, imaging revealed a lesion 7.2 x 5.6 x 4.5 cm in size involving the cervix. The decision was made to proceed with surgery due to the lack of response to treatment. This patient had a total abdominal hysterectomy and bilateral salpingo-oophorectomy with no evidence of recurrent disease four months after surgery. The authors recommend primary surgery for cases of carcinosarcoma, as many cases are unresponsive to chemoradiation [12].

Another case report was conducted by Montero-Macias et al., which focused on a 49-year-old female with FIGO stage IIIC2 locally advanced undifferentiated cervical cancer [13]. This patient had a 9.1 cm mass that involved her cervix, uterus, uterine serosa, parametrium, and left pelvic wall, and she developed a left hydronephrosis. A cervical biopsy was consistent with a squamous cell carcinoma that was HPV 16 positive. This patient received two cycles of capecitabine/cisplatin and radiation therapy to a total dose of 6480 cGy in 36 fractions to the tumor with eight cycles of cisplatin. The patient had a partial response to treatment at the three-month follow-up. With only a partial response, the decision was made to offer additional therapy consisting of carboplatin, paclitaxel, plus bevacizumab [13]. Following the completion of this regimen, the tumor size decreased by half, and a simple hysterectomy was performed. At one year follow-up, there was no evidence of recurrence [13].

Additional studies to support combined modality treatment include a meta-analysis of individual patient data from 13 randomized trials. This study revealed an absolute survival benefit of 6% and a disease-free survival benefit of 8% over five years with concurrent chemoradiotherapy [14]. However, a decreasing benefit was seen in the effect of chemoradiotherapy on survival with increasing tumor stage, with only a 3% difference seen in stage III to stage IVA at five years [14].

Shrivastava and colleagues reported similar findings in the cisplatin chemoradiation versus radiation alone in FIGO stage IIIB squamous cell carcinoma of the uterine cervix [15]. This single-institution randomized clinical trial demonstrated a statistically significant improved disease-free survival and overall survival in women with stage IB cervical cancer treated with concurrent chemoradiation. The study revealed an absolute benefit of 8.5% in disease-free survival and an 8% benefit in overall survival [15].

A second incidence peak of cervical cancer appears between 60 and 70 years old, although the average age of the patients at diagnosis is about 53 years old [16]. In elderly patients over 65 with cervical cancer, the five-year survival rate is reported at 40.8%. Many older women with cervical cancer tend to be weaker and have multiple comorbidities, such as diabetes or cardiac disease, which can make treatment more difficult to tolerate. Patients over 65 years of age are also under-represented in clinical trials. Because of this, patients greater than 65 years of age tend not to be treated aggressively, which can lead to a worse local control and survival outcome compared with younger patients [16].

Finally, a study by Albert et al. found that older patients with cervical cancer received less aggressive treatment and had lower overall survival compared with younger patients. Age was found to be an independent predictor for the receipt of standard-of-care treatment for cervical cancer [17]. On multivariable analysis, ages 71-80 and age 80 were associated with a decrease in overall survival. A subgroup analysis was performed for patients with significant comorbidities and treatment with standard of care resulted in increased five-year OS as compared to incomplete treatment [17].

## Conclusions

Cervical cancer that involves the entire pelvis in a patient greater than 65 years of age is a rare entity. This case report is unique, given the size of the tumor lesion, the age of our patient, the advanced stage of the disease, and the tumor's complete radiographic response to treatment with no disease noted on MRI before brachytherapy. Even though patients greater than 65 years old with cervical cancer may seem incurable at initial presentation, secondary to locally advanced disease, large tumor size, and stage III or IV disease, a dramatic response to treatment and possible long-term control and cure can be obtained with radiation and chemotherapy. Based on our patient and a review of the literature, patients with FIGO stage IIIB cervical cancer who are greater than 65 years of age and who are overall fit should be considered for a combination of chemotherapy and radiation, including brachytherapy with intent for cure.
